# 4-Hydr­oxy-3-meth­oxy-5-nitro­aceto­phenone (5-nitro­apocynin)

**DOI:** 10.1107/S160053680903390X

**Published:** 2009-08-29

**Authors:** Sainath Babu, Achuthan C. Raghavamenon, Frank R. Fronczek, Rao M. Uppu

**Affiliations:** a Department of Environmental Toxicology and the Health Research Center, Southern University and A&M College, Baton Rouge, LA 70813, USA; b Department of Chemistry, Louisiana State University, Baton Rouge, LA 70803-1804, USA

## Abstract

The title mol­ecule, C_9_H_9_NO_5_, is close to planar (r.m.s. deviation from the mean plane of the non-H atoms = 0.058 Å). The OH group forms a bifurcated O—H⋯(O,O) hydrogen bond, with the intra­molecular component to a nitro O atom and the inter­molecular component to a keto O atom, the latter resulting in chains along [20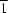]. A C—H⋯O inter­action reinforces the packing.

## Related literature

For medicinal background, see: Gernapudi *et al.* (2009 ▶); Geronikaki & Gavalas (2006 ▶); Hayashi *et al.* (2005 ▶); Heumuller *et al.* (2008 ▶); Matés *et al.* (2009 ▶); Muijsers *et al.* (2001 ▶); Sawa *et al.* (2000 ▶); Schopfer *et al.* (2003 ▶); Stefanska & Pawliczak (2008 ▶); Stolk *et al.* (1994 ▶); Tajik *et al.* (2009 ▶); Thomas *et al.* (2002 ▶); Touyz (2008 ▶); Ximenes *et al.* (2007 ▶).
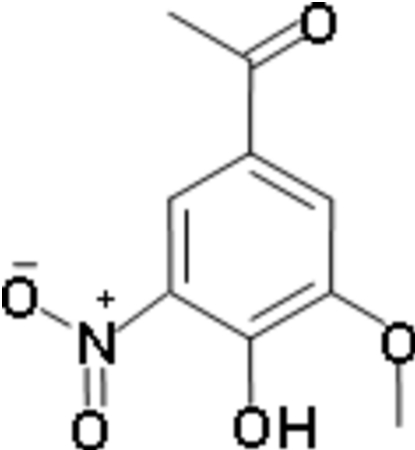


## Experimental

### 

#### Crystal data

C_9_H_9_NO_5_
*M* *_r_* = 211.17
Monoclinic, 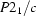

*a* = 6.6598 (10) Å
*b* = 16.815 (2) Å
*c* = 8.0491 (11) Å
β = 96.485 (7)°
*V* = 895.6 (2) Å^3^
*Z* = 4
Mo *K*α radiation
μ = 0.13 mm^−1^
*T* = 90 K
0.40 × 0.30 × 0.15 mm

#### Data collection

Nonius KappaCCD diffractometer with Oxford Cryostream
Absorption correction: none
22479 measured reflections
4255 independent reflections
3226 reflections with *I* > 2σ(*I*)
*R*_int_ = 0.025

#### Refinement

*R*[*F* ^2^ > 2σ(*F* ^2^)] = 0.042
*wR*(*F* ^2^) = 0.120
*S* = 1.04
4255 reflections
147 parameters
H atoms treated by a mixture of independent and constrained refinement
Δρ_max_ = 0.62 e Å^−3^
Δρ_min_ = −0.32 e Å^−3^

### 

Data collection: *COLLECT* (Nonius, 2000 ▶); cell refinement: *SCALEPACK* (Otwinowski & Minor, 1997 ▶); data reduction: *DENZO* (Otwinowski & Minor, 1997 ▶) and *SCALEPACK*; program(s) used to solve structure: *SIR97* (Altomare *et al.*, 1999 ▶); program(s) used to refine structure: *SHELXL97* (Sheldrick, 2008 ▶); molecular graphics: *ORTEP-3 for Windows* (Farrugia, 1997 ▶); software used to prepare material for publication: *SHELXL97*.

## Supplementary Material

Crystal structure: contains datablocks global, I. DOI: 10.1107/S160053680903390X/hb5058sup1.cif

Structure factors: contains datablocks I. DOI: 10.1107/S160053680903390X/hb5058Isup2.hkl

Additional supplementary materials:  crystallographic information; 3D view; checkCIF report

## Figures and Tables

**Table 1:** Hydrogen-bond geometry (Å, °)

*D*—H⋯*A*	*D*—H	H⋯*A*	*D*⋯*A*	*D*—H⋯*A*
O1—H1*O*⋯O4	0.878 (14)	1.850 (15)	2.5939 (10)	141.3 (13)
O1—H1*O*⋯O3^i^	0.878 (14)	2.271 (14)	2.8660 (9)	124.9 (12)
C2—H2⋯O4^ii^	0.952 (12)	2.439 (12)	3.3831 (12)	171.4 (11)

Symmetry codes: (i) 
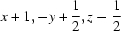
; (ii) 
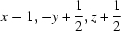
.

**Fig. 1.**
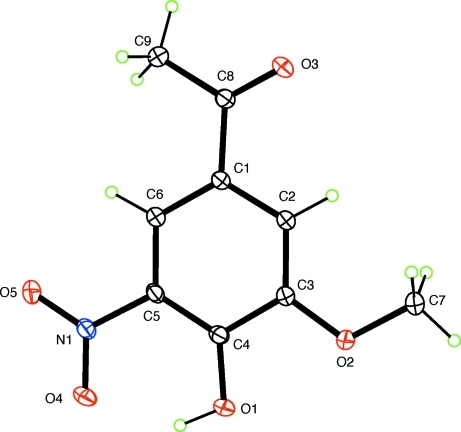
The molecular structure of (I): ellipsoids at the 50% level, with H atoms having arbitrary radius.

**Fig. 2.**
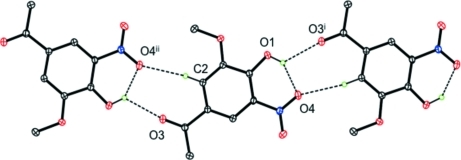
A portion of the hydrogen-bonded chain, showing the bifurcated hydrogen bond and accompanying C–H···O interaction; (ii) x - 1, 1/2 - y, 1/2 + z.

**Fig. 3.**
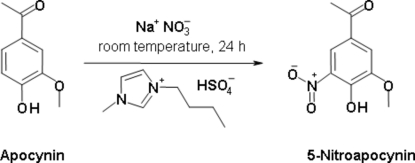
Nitration of apocynin

**Fig. 4.**
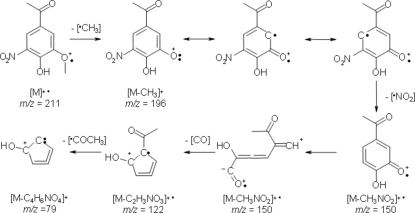
A proposed route of mass spectrometry-electron ionization (70 eV) fragmentation of 4-hydroxy-3-methoxy-5-nitroacetophenone.

## supplementary crystallographic information

### Comment

The growing concern of multiple side effects associated with the use of steroidal anti-inflammatory drugs has led investigators to explore alternative and more natural remedies to counter oxidative stress in cancer and other degenerative diseases (Geronikaki & Gavalas, 2006; Matés *et al.*, 2009). Apocynin (4-hydroxy-3-methoxy-acetophenone), also called acetovanillone, isolated from plants belonging to the apocyanaceae family (*e.g.*, *Apocynum cannabinum*) seems to be a promising drug (or prodrug) that can be effective in various inflammatory conditions (Hayashi *et al.*, 2005; Muijsers *et al.*, 2001; Stefanska & Pawliczak, 2008). For a long time, apocynin has been thought to inhibit plasma membrane NADPH oxidase activity by interfering with the assembly of its cytosolic components, p40, p47, and p67 (Stolk *et al.*, 1994). This view of a direct action of apocynin on the NADPH oxidase system has been challenged in recent years (Heumuller *et al.*, 2008). Suggestions have been made that apocynin requires metabolic activation to diapocynin (DiApo), presumably involving intracellular peroxidase(*s*) (Touyz, 2008; Ximenes *et al.*, 2007). In a recent study, we showed that apocynin readily reacts with free radicals of carbonate (CO_3_^.-^) and nitrogen dioxide (^.^NO_2_) formed in reactions of peroxynitrite (PN) with CO_2_, resulting in the formation of 5-nitroapocynin and DiApo as major products (Gernapudi *et al.*, 2009). Based on these observations, it has been suggested that a detailed study of the oxidative transformation of apocynin and its derivates by PN/CO_2_ and possibly other oxidative, nitrative and/or nitrosative systems (Sawa *et al.*, 2000; Schopfer *et al.*, 2003; Thomas *et al.*, 2002) would be necessary to provide a template for screening of antioxidant activity and a module that could help in the design of effective inhibitors of the NADPH oxidase system. Towards this end, we have synthesized 5-nitroapocynin using sodium nitrate in combination with an acidic ionic liquid, 1-butyl-3-methylimidazolium hydrogen sulfate ([bmim] [HSO_4_]), in CH_3_CN solvent at room temperature (Fig. 3).

The molecule is shown in Fig. 1. The phenyl ring is essentially planar, with RMS deviation 0.0046 Å and maximum deviation 0.0069 (6) Å for C5. The substituents are twisted only slightly out of the phenyl plane, as described in the Abstract. Figure 2 shows the hydrogen bonding pattern, in which the OH group forms both an intramolecular interaction and a much less linear intermolecular interaction. These are described in Table 2, and form a chain in the [201] direction. The intermolecular component is accompanied by a near-linear C2–H2···O4 (at *x* - 1, 1/2 - *y*, 1/2 + *z*) interaction, having C···O distance 3.3831 (12) Å, H···O distance 2.439 (12) Å, and angle 171.4 (11)° about H. The N1–O4 bond, 1.2438 (10) Å, to the O atom involved in the intramolecular hydrogen bond, is slightly longer than the other, N1–O5, 1.2255 (10) Å.

### Experimental

Chemicals and solvents used in the synthesis and recrystallization were obtained as follows: apocynin, [bmim] [HSO_4_], and sodium nitrate from Sigma (St. Louis, MO) and acetonitrile and hexane from Mallinckrodt (Phillipsburg, NJ). Water used was ultrapure with resistance ≥18.2 *M*Ω/cm.

Nitration of apocynin (Fig. 3) was performed according to the method of Tajik and colleagues (Tajik *et al.*, 2009) with some modifications. Briefly, to 3.32 g (20 mmol) of apocynin in 80 ml of CH_3_CN was added 5.72 g (20 mmol) of [bmim] [HSO_4_] and 1.7 g (20 mmol) of NaNO_3_, and the mixture was stirred at room temperature. Aliquots (0. 1 ml each) of the reaction mixture, drawn at various time points, were diluted 100–500-fold with 0.1 N NaOH and measured photometrically at 410 nm. When the absorbance at 410 nm reached a maximum (*i.e.*, typically after 24 h), the reaction mixture was filtered and the filtrate evaporated under low pressure (200 mm H g) with mild heating (50°C or slightly higher). The thick brown liquid-like residue was extracted with hot hexane and recrystallized twice. The compound resolved as a single peak (retention time = 11.507 min) on Varian VF-5MS capillary column (30-m length, 0.25-mm internal diameter, 0.25-µm film thickness) with helium as the carrier gas at a flow of 1 ml. min^-1^ (injection port, 250°C; oven, 60°C for 5 min (isothermal); 20°C min^-1^ up to 230°C (ramp), and held at 230 °C for 18.5 min (isothermal); split, 25:1). The ion chromatogram of the peak eluting at 11.507 min showed a molecular ion [*M*]^+^ at m/*z* 211 (31%; relative to the base peak) and other fragments at m/*z* values of 196 (100%; base peak; [M—CH_3_]^+^), 150 (23%; [M—CH_3_NO_2_]^+.^), 122 (11%; [M—C_2_H_3_NO_3_]^+.^) and 79 (6%; [M—C_4_H_6_NO_4_]^+.^) (Fig. 4). Single crystals of (I) in the form of golden-yellow needles were grown from methanol.

### Refinement

H atoms on C were located from difference maps, and their coordinates were refined, except for those on methyl groups, which were idealized with C—H distance 0.98 Å. A torsional parameter was refined for each methyl group. *U*_iso_ for H were assigned as 1.2 times *U*_eq_ of the attached atoms (1.5 for methyl). The top ten difference map peaks lie on bonds, the largest at the midpoint of C3—C4, 0.71 Å from C4.

### Crystal data

**Table:**

C_9_H_9_NO_5_	*F*(000) = 440
*M**_r_* = 211.17	*D*_x_ = 1.566 Mg m^−^^3^
Monoclinic, *P*2_1_/*c*	Mo *K*α radiation, λ = 0.71073 Å
Hall symbol: -P 2ybc	Cell parameters from 4183 reflections
*a* = 6.6598 (10) Å	θ = 2.5–36.3°
*b* = 16.815 (2) Å	µ = 0.13 mm^−^^1^
*c* = 8.0491 (11) Å	*T* = 90 K
β = 96.485 (7)°	Needle fragment, golden yellow
*V* = 895.6 (2) Å^3^	0.40 × 0.30 × 0.15 mm
*Z* = 4	

### Data collection

**Table:**

Nonius KappaCCD diffractometer with Oxford Cryostream	3226 reflections with *I* > 2σ(*I*)
Radiation source: fine-focus sealed tube	*R*_int_ = 0.025
graphite	θ_max_ = 36.3°, θ_min_ = 2.8°
ω and φ scans	*h* = −10→10
22479 measured reflections	*k* = −28→26
4255 independent reflections	*l* = −13→13

### Refinement

**Table:**

Refinement on *F*^2^	Primary atom site location: structure-invariant direct methods
Least-squares matrix: full	Secondary atom site location: difference Fourier map
*R*[*F*^2^ > 2σ(*F*^2^)] = 0.042	Hydrogen site location: inferred from neighbouring sites
*wR*(*F*^2^) = 0.120	H atoms treated by a mixture of independent and constrained refinement
*S* = 1.04	*w* = 1/[σ^2^(*F*_o_^2^) + (0.0585*P*)^2^ + 0.2019*P*] where *P* = (*F*_o_^2^ + 2*F*_c_^2^)/3
4255 reflections	(Δ/σ)_max_ < 0.001
147 parameters	Δρ_max_ = 0.62 e Å^−^^3^
0 restraints	Δρ_min_ = −0.32 e Å^−^^3^

### Special details

**Table:**

Geometry. All e.s.d.'s (except the e.s.d. in the dihedral angle between two l.s. planes) are estimated using the full covariance matrix. The cell e.s.d.'s are taken into account individually in the estimation of e.s.d.'s in distances, angles and torsion angles; correlations between e.s.d.'s in cell parameters are only used when they are defined by crystal symmetry. An approximate (isotropic) treatment of cell e.s.d.'s is used for estimating e.s.d.'s involving l.s. planes.
Refinement. Refinement of *F*^2^ against ALL reflections. The weighted *R*-factor *wR* and goodness of fit *S* are based on *F*^2^, conventional *R*-factors *R* are based on *F*, with *F* set to zero for negative *F*^2^. The threshold expression of *F*^2^ > σ(*F*^2^) is used only for calculating *R*-factors(gt) *etc*. and is not relevant to the choice of reflections for refinement. *R*-factors based on *F*^2^ are statistically about twice as large as those based on *F*, and *R*- factors based on ALL data will be even larger.

### Fractional atomic coordinates and isotropic or equivalent isotropic displacement parameters (Å^2^)

**Table:**

	*x*	*y*	*z*	*U*_iso_*/*U*_eq_	
O1	0.70859 (9)	0.17341 (4)	0.57565 (8)	0.01682 (13)	
H1O	0.805 (2)	0.1965 (9)	0.5277 (18)	0.025*	
O2	0.40131 (9)	0.12263 (4)	0.70993 (9)	0.01727 (13)	
O3	0.03482 (9)	0.36827 (4)	0.88871 (9)	0.01850 (14)	
O4	0.90549 (10)	0.29612 (4)	0.48481 (9)	0.02148 (15)	
O5	0.81333 (10)	0.41618 (4)	0.53665 (9)	0.02082 (14)	
N1	0.79036 (11)	0.34401 (4)	0.54412 (9)	0.01483 (13)	
C1	0.32973 (11)	0.33924 (5)	0.76315 (10)	0.01274 (14)	
C2	0.29375 (12)	0.25663 (5)	0.77082 (10)	0.01340 (14)	
H2	0.1831 (18)	0.2368 (8)	0.8237 (15)	0.016*	
C3	0.42194 (12)	0.20294 (5)	0.70775 (10)	0.01312 (14)	
C4	0.59325 (12)	0.22979 (5)	0.63245 (10)	0.01309 (14)	
C5	0.62316 (11)	0.31230 (5)	0.62424 (10)	0.01315 (14)	
C6	0.49407 (12)	0.36689 (5)	0.68977 (10)	0.01365 (14)	
H6	0.5227 (18)	0.4219 (8)	0.6815 (15)	0.016*	
C7	0.22582 (13)	0.09248 (5)	0.77728 (12)	0.01791 (16)	
H7A	0.2325	0.1066	0.8959	0.027*	
H7B	0.2211	0.0345	0.7654	0.027*	
H7C	0.1041	0.1158	0.7165	0.027*	
C8	0.18762 (12)	0.39440 (5)	0.83692 (10)	0.01404 (14)	
C9	0.23465 (14)	0.48181 (5)	0.84358 (12)	0.01944 (17)	
H9A	0.2105	0.5043	0.7308	0.029*	
H9B	0.3766	0.4897	0.8876	0.029*	
H9C	0.1476	0.5084	0.9167	0.029*	

### Atomic displacement parameters (Å^2^)

**Table:**

	*U*^11^	*U*^22^	*U*^33^	*U*^12^	*U*^13^	*U*^23^
O1	0.0147 (3)	0.0152 (3)	0.0220 (3)	0.0018 (2)	0.0084 (2)	−0.0013 (2)
O2	0.0163 (3)	0.0115 (3)	0.0255 (3)	−0.0003 (2)	0.0087 (2)	0.0008 (2)
O3	0.0156 (3)	0.0183 (3)	0.0233 (3)	−0.0003 (2)	0.0094 (2)	−0.0002 (2)
O4	0.0179 (3)	0.0203 (3)	0.0287 (3)	−0.0003 (2)	0.0136 (3)	−0.0035 (3)
O5	0.0232 (3)	0.0155 (3)	0.0257 (3)	−0.0043 (2)	0.0110 (3)	0.0008 (2)
N1	0.0137 (3)	0.0163 (3)	0.0152 (3)	−0.0018 (2)	0.0047 (2)	−0.0004 (2)
C1	0.0120 (3)	0.0131 (3)	0.0137 (3)	0.0005 (2)	0.0036 (2)	0.0006 (2)
C2	0.0123 (3)	0.0135 (3)	0.0150 (3)	−0.0002 (2)	0.0042 (2)	0.0007 (3)
C3	0.0124 (3)	0.0126 (3)	0.0148 (3)	−0.0004 (2)	0.0033 (2)	0.0009 (3)
C4	0.0118 (3)	0.0144 (3)	0.0135 (3)	0.0008 (2)	0.0033 (2)	−0.0004 (3)
C5	0.0111 (3)	0.0152 (3)	0.0139 (3)	−0.0015 (2)	0.0044 (2)	0.0002 (3)
C6	0.0133 (3)	0.0135 (3)	0.0147 (3)	−0.0003 (2)	0.0038 (2)	0.0005 (3)
C7	0.0172 (3)	0.0152 (3)	0.0223 (4)	−0.0020 (3)	0.0065 (3)	0.0023 (3)
C8	0.0136 (3)	0.0146 (3)	0.0145 (3)	0.0013 (2)	0.0039 (3)	0.0012 (3)
C9	0.0201 (4)	0.0134 (3)	0.0264 (4)	0.0013 (3)	0.0095 (3)	0.0010 (3)

### Geometric parameters (Å, °)

**Table:**

O1—C4	1.3330 (10)	C2—H2	0.952 (12)
O1—H1O	0.878 (14)	C3—C4	1.4245 (11)
O2—C3	1.3576 (10)	C4—C5	1.4043 (12)
O2—C7	1.4352 (11)	C5—C6	1.4008 (11)
O3—C8	1.2240 (10)	C6—H6	0.948 (13)
O4—N1	1.2438 (10)	C7—H7A	0.9800
O5—N1	1.2255 (10)	C7—H7B	0.9800
N1—C5	1.4499 (10)	C7—H7C	0.9800
C1—C6	1.3817 (11)	C8—C9	1.5026 (12)
C1—C2	1.4120 (12)	C9—H9A	0.9800
C1—C8	1.4952 (11)	C9—H9B	0.9800
C2—C3	1.3783 (11)	C9—H9C	0.9800
			
C4—O1—H1O	108.5 (10)	C4—C5—N1	120.29 (7)
C3—O2—C7	116.37 (7)	C1—C6—C5	119.34 (8)
O5—N1—O4	122.44 (7)	C1—C6—H6	122.2 (7)
O5—N1—C5	119.50 (7)	C5—C6—H6	118.4 (7)
O4—N1—C5	118.06 (7)	O2—C7—H7A	109.5
C6—C1—C2	119.75 (7)	O2—C7—H7B	109.5
C6—C1—C8	121.91 (7)	H7A—C7—H7B	109.5
C2—C1—C8	118.34 (7)	O2—C7—H7C	109.5
C3—C2—C1	120.87 (7)	H7A—C7—H7C	109.5
C3—C2—H2	118.5 (8)	H7B—C7—H7C	109.5
C1—C2—H2	120.6 (8)	O3—C8—C1	120.03 (8)
O2—C3—C2	125.38 (7)	O3—C8—C9	121.13 (8)
O2—C3—C4	114.07 (7)	C1—C8—C9	118.83 (7)
C2—C3—C4	120.55 (7)	C8—C9—H9A	109.5
O1—C4—C5	126.60 (7)	C8—C9—H9B	109.5
O1—C4—C3	116.15 (7)	H9A—C9—H9B	109.5
C5—C4—C3	117.24 (7)	C8—C9—H9C	109.5
C6—C5—C4	122.23 (7)	H9A—C9—H9C	109.5
C6—C5—N1	117.47 (7)	H9B—C9—H9C	109.5
			
C6—C1—C2—C3	0.78 (12)	C3—C4—C5—N1	−177.86 (7)
C8—C1—C2—C3	−178.74 (7)	O5—N1—C5—C6	0.14 (12)
C7—O2—C3—C2	2.89 (12)	O4—N1—C5—C6	−179.69 (7)
C7—O2—C3—C4	−177.15 (7)	O5—N1—C5—C4	179.34 (8)
C1—C2—C3—O2	179.60 (8)	O4—N1—C5—C4	−0.48 (12)
C1—C2—C3—C4	−0.36 (12)	C2—C1—C6—C5	−0.15 (12)
O2—C3—C4—O1	−0.18 (10)	C8—C1—C6—C5	179.35 (7)
C2—C3—C4—O1	179.78 (7)	C4—C5—C6—C1	−0.92 (12)
O2—C3—C4—C5	179.38 (7)	N1—C5—C6—C1	178.27 (7)
C2—C3—C4—C5	−0.66 (12)	C6—C1—C8—O3	174.11 (8)
O1—C4—C5—C6	−179.19 (8)	C2—C1—C8—O3	−6.38 (12)
C3—C4—C5—C6	1.31 (12)	C6—C1—C8—C9	−5.00 (12)
O1—C4—C5—N1	1.65 (13)	C2—C1—C8—C9	174.51 (8)

### Hydrogen-bond geometry (Å, °)

**Table:**

*D*—H···*A*	*D*—H	H···*A*	*D*···*A*	*D*—H···*A*
O1—H1O···O4	0.878 (14)	1.850 (15)	2.5939 (10)	141.3 (13)
O1—H1O···O3^i^	0.878 (14)	2.271 (14)	2.8660 (9)	124.9 (12)
C2—H2···O4^ii^	0.952 (12)	2.439 (12)	3.3831 (12)	171.4 (11)

Symmetry codes: (i) *x*+1, −*y*+1/2, *z*−1/2; (ii) *x*−1, −*y*+1/2, *z*+1/2.
